# Gray and White Matter Demyelination and Remyelination Detected with Multimodal Quantitative MRI Analysis at 11.7T in a Chronic Mouse Model of Multiple Sclerosis

**DOI:** 10.3389/fnins.2016.00491

**Published:** 2016-10-27

**Authors:** Alexandra Petiet, Marie-Stéphane Aigrot, Bruno Stankoff

**Affiliations:** ^1^Center for Neuroimaging Research, Brain and Spine InstituteParis, France; ^2^Pierre and Marie Curie University/INSERM UMR975, Brain and Spine InstituteParis, France; ^3^Department of Neurology, Saint-Antoine Hospital, Assistance Publique-Hôpitaux de Paris (AP-HP)Paris, France

**Keywords:** multiple sclerosis, demyelination, remyelination, cuprizone, T_2_, diffusion

## Abstract

Myelin is a component of the nervous system that is disrupted in multiple sclerosis, resulting in neuro-axonal degeneration. The longitudinal effect of chronic cuprizone-induced demyelination was investigated in the cerebral gray and white matter of treated mice and the spontaneous remyelination upon treatment interruption. Multimodal Magnetic Resonance Imaging and a Cryoprobe were used at 11.7T to measure signal intensity ratios, T_2_ values and diffusion metrics. The results showed significant and reversible modifications in white matter and gray matter regions such as in the rostral and caudal corpus callosum, the external capsule, the cerebellar peduncles, the caudate putamen, the thalamus, and the somatosensory cortex of treated mice. T_2_ and radial diffusivity metrics appeared to be more sensitive than fractional anisotropy, axial diffusivity or mean diffusivity to detect those cuprizone-induced changes. In the gray matter, only signal and T_2_ metrics and not diffusion metrics were sensitive to detect any changes. Immunohistochemical qualitative assessments in the same regions confirmed demyelination and remyelination processes. These multimodal data will provide better understanding of the dynamics of cuprizone-induced de- and remyelination in white and gray matter structures, and will be the basis to test therapies in experimental models.

## Introduction

The myelin sheath is an essential component of the vertebrate nervous system enabling an accelerated conduction of nerve impulses together with reduced energy consumption. In the human central nervous system, several debilitating diseases are secondary to myelin damage, the most frequent being multiple sclerosis (MS), where recurrent episodes of demyelination result in neuro-axonal degeneration. Promoting myelin repair, an endogenous process that was shown to fail as disease progresses, is a crucial therapeutic challenge in MS. Not only should it allow restoration of normal conduction and functional recovery, but it may also prevent axonal and neuronal degeneration. Such a neuroprotective role of myelin could be particularly important in the cortical gray matter (GM) where myelin sheaths, despite being less abundant, could play a crucial role in neuronal survival (Kang et al., 2013). Interestingly the existence of GM demyelinating cortical lesions in the brains of MS subjects, which are now known to be an important hallmark of MS (Kidd et al., 1999; Kutzelnigg et al., 2005; Nelson et al., 2008; Geurts et al., 2011; Mike et al., 2011; Staugaitis et al., 2012), has recently been demonstrated. Such cortical demyelination is thought to appear during the first stages of the disease, but subsequently becomes increasingly pronounced during the later phases. This process could be a contributing factor in disability among MS patients. In addition, efficient remyelinating abilities have been observed in such lesions, with an even higher efficiency than in white matter (WM) lesions (Chang et al., 2012).

The cuprizone (CPZ) mouse model is frequently used to recapitulate demyelination that occurs in MS. Cuprizone is a copper chelator (biscyclohexanone oxaldihydrazone) that causes cell death of the oligodendrocytes, leading to demyelination (see review by Torkildsen et al., 2008). This neurotoxin has been used in mice since 1966 and is administered in food (Carlton, 1966). Mice fed with CPZ were initially described as presenting WM demyelination mainly in the corpus callosum (CC) and in the cerebellar peduncles (CP) (Suzuki and Kikkawa, 1969; Matsushima and Morell, 2001). Recent investigations have pointed that CPZ treated mice were also characterized by an extended GM demyelination occurring in the cerebellar cortex (Skripuletz et al., 2010), in the deep GM, and in the cerebral cortex (Skripuletz et al., 2008, 2011; Xiao et al., 2008). A key feature of the CPZ model is that following withdrawal of the toxin, remyelination takes place spontaneously with a high level of efficacy both in WM and GM areas, especially when a short-duration CPZ exposure paradigm is applied, i.e., 5–6-week treatment (TX) (Skripuletz et al., 2011). When the TX is prolonged for 12 weeks, the remyelination process is delayed and only partly efficient (Armstrong et al., 2006; Skripuletz et al., 2008; Lindner et al., 2009). Overall the CPZ model enables the investigation of the mechanisms of remyelination both in the WM and in the GM, either in optimal or deficient conditions. The development of repair therapies requires methods to monitor myelin dynamics in living individuals. Magnetic Resonance Imaging (MRI) is widely used to study brain disorders. Conventional structural imaging using T_2_ contrast easily enables the detection of macroscopic WM lesions, however it is not specific of a particular biological abnormality, and generally fails to identify cortical demyelination due to weak spatial resolution and sparse cortical network of fibers in the cortex resulting in poor contrast for myelin. Diffusion-weighted MRI is based on the diffusion of water molecules, which can be restricted by membranes, and can thereby provide differential measurements of myelin and axonal integrity (Song et al., 2002; Bodini et al., 2015). Several MRI studies attempted to visualize demyelination and remyelination in the WM of mice treated with CPZ using different MRI techniques such as T_2_-weighted imaging, which showed enhanced signal in demyelinated regions (Yu et al., 2004; Merkler et al., 2005; Thiessen et al., 2013), diffusion-weighted imaging (Song et al., 2005; Sun et al., 2006; Wu et al., 2008; Boretius et al., 2012; Zhang et al., 2012) showing increased radial diffusivity (RD) and decreased fractional anisotropy (FA) in demyelinated regions, or magnetization transfer imaging (Zaaraoui et al., 2008; Turati et al., 2015) showing decreased magnetization transfer ratios after demyelination and recovery after remyelination. More recently, WM tract integrity metrics derived from diffusion kurtosis imaging have been shown to be more specific to assess the WM microstructural changes in the CPZ model (Jelescu et al., 2016).

However, most of these studies have focused on the CC, and none has explored the dynamics of GM pathology. We used ultra-high-field MRI and a CryoProbe™ in order to provide increased signal-to-noise ratio (SNR) and resolution to investigate both GM and WM alterations over 7 brain regions during TX and recovery in the chronic CPZ mouse model.

## Materials and methods

### Animal model

All animal experiments were performed in accordance with the European Council Directive (88/609/EEC).

We used C57Bl/6J PLP-GFP L42 transgenic mice, where the fluorescent GFP was spontaneously expressed in myelin. Six 8-week old female mice were fed with 0.2% CPZ and imaged before TX and 12 weeks after TX. The animals were then fed normal chow during 12 additional weeks and weighed on a daily basis. They were imaged at 6 weeks (*n* = 5) and 12 weeks (*n* = 4) without TX to monitor recovery. One mouse was sacrificed at 12 weeks of TX and one mouse at 12 weeks of recovery for immunohistochemical assessment.

Throughout all imaging sessions, the animals were anesthetized with 1–1.5% isoflurane (Abbott Animal Health) mixed with oxygen (1:5 O_2_:air) delivered through a nose cone at a flow rate of 1 L/min. Physiological parameters were recorded via a monitoring system (S.A. Instruments Inc.). The temperature of the animals was maintained at 37°C through a circulating heated water system, and measured with a rectal probe. The respiration of the animals was monitored using a pressure pad positioned under their abdomen.

### Data acquisition

All images were acquired with an 11.7-T system (Bruker Biospec 117/16 USR horizontal bore, 750 mT/m gradients, Paravision 5.1, Ettlingen, Germany) and a helium-cooled ^1^H quadrature transmit-receive surface CryoProbe™ for mouse head (Bruker, Ettlingen, Germany). The receiver gain was manually adjusted to optimize the gain over the upper 3/4 of the brain. High-resolution anatomical T_2_-weighted (T_2_w) images were acquired with a 2D rapid acquisition with relaxation enhanced sequence; repetition time (*TR*) = 6000 ms; echo time (*TE*) = 40 ms; Matrix = 256 × 256; field-of-view (FOV) = 15.36 × 15.36 mm^2^; resolution = 60 × 60 μm^2^; slice thickness = 220 μm; number of excitations = 1; acquisition time = 17 min. Parametric T_2_ maps were obtained from a multi-slice multi-echo sequence with *TR* = 5500 ms; *TE* = 15–120 ms/5-ms increments; Matrix = 128 × 128; FOV = 12.8 × 12.8 mm^2^ (resolution = 100 × 100 μm^2^); slice thickness = 200 μm; number of excitations = 2; acquisition time = 15 min. Three-dimensional diffusion echo-planar images were acquired with *TR* = 500 ms; *TE* = 20 ms; Matrix = 160 × 96 × 32; FOV = 24 × 14.4 × 9.6 mm^3^ (resolution = 150 × 150 × 300 μm^3^); 46 directions; *b* = 1000 s/mm^2^; δ = 4 ms; Δ = 10 ms; acquisition time = 41 min.

### Data analysis

The T_2_w images were coregistered using the Linear Image Registration Tool from the Oxford Center for Functional MRI of the Brain (Jenkinson and Smith, 2001; Jenkinson et al., 2002). Signal ratios (Sr) were calculated from the coregistered images between the signal in manually-drawn regions of interest (ROIs) in the GM or WM and the signal from the cerebrospinal fluid as done by Yu et al. (2004) and Chandran et al. (2012), using the Image Processing and Analysis in Java software (http://imagej.nih.gov). T_2_ values were calculated from a pixel-wise regression fit function from the multi-echo sequence using the PV5.1 image sequence analysis tool package. Diffusion parametric maps were also generated from the Paravision 5.1 package. Diffusion metrics such as FA, AD (as a marker of axonal damage), RD (as a marker of myelin damage) and mean diffusivity (MD) were measured.

**Figure 1:**
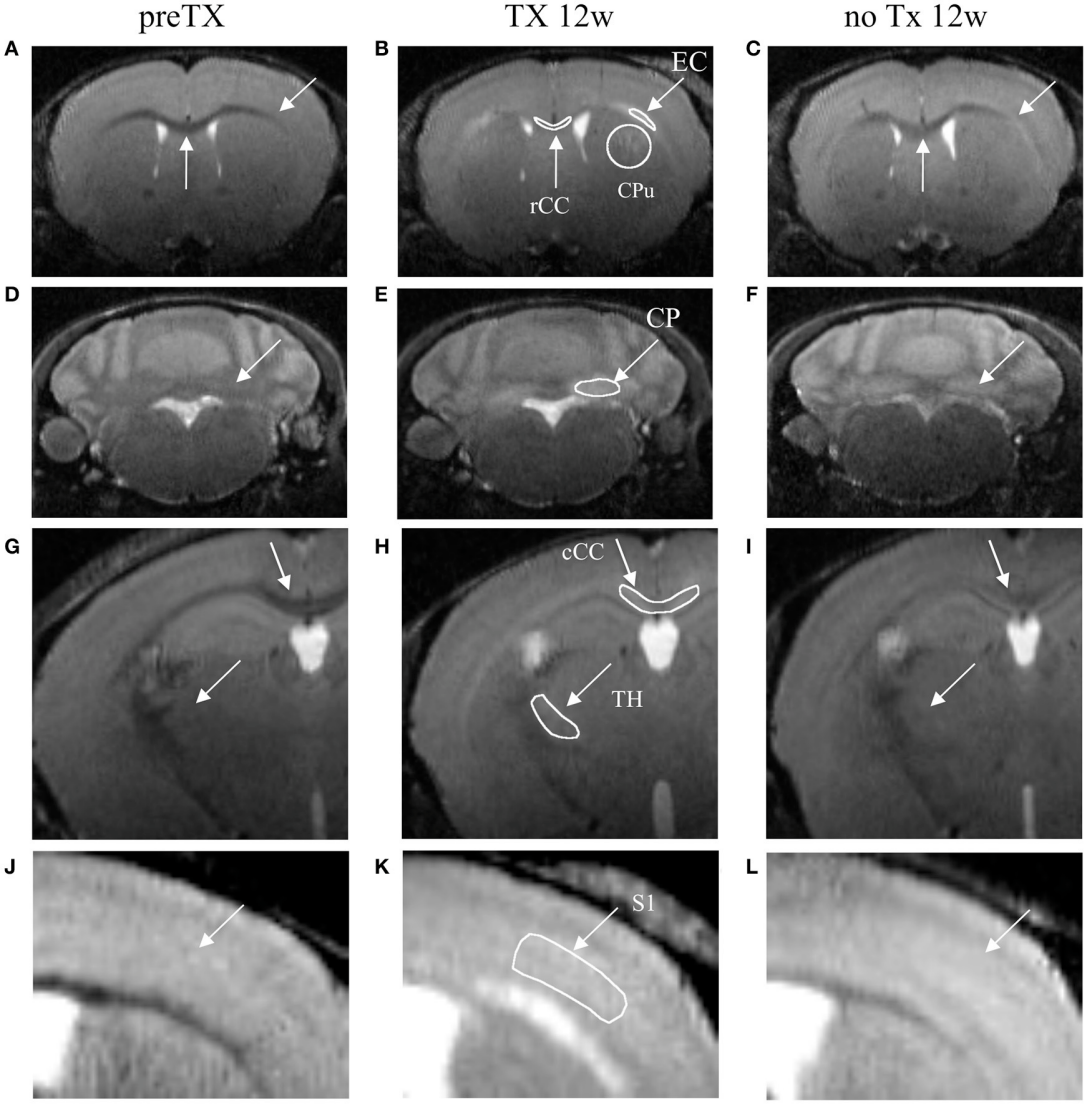
**T_2_-weighted coronal images of the dynamics of demyelination/remyelination over the course of the pre-TX (A,D,G,J), 12w TX (B,E,H,K) and 12w no TX phases (C,F,I,L)**. Hyperintensities were detected after 12 weeks (12w) of TX in CPu, EC, and rCC **(B)**; in CP **(E)**; in TH and cCC **(H)**; and in S1 **(K)**. After 12 weeks without TX **(C,F,I,L)**, the signal intensities decreased back. Manually-drawn regions of interest are shown in the middle panel in white.

Anatomical landmarks were used to position the ROIs at the same place in all mice (Figure 1). The ROIs included the rostral and caudal CC (rCC and cCC), the external capsule (EC), the primary somatosensory cortex (S1), the cerebellar peduncles (CP), the dorsal caudate putamen (CPu) and the ventral posteromedial and posterolateral thalamic nuclei (TH). Measurements for a given ROI were averaged from the left and the right hemispheres over 3 adjacent slices.

To compare the experimental groups at different time points, the statistical analysis was performed using the Matlab mutlcompare function for pairwise multiple comparison (Matlab 7.11.0 R2010b, Statistics Toolbox). The results were considered statistically significant when the comparison intervals were disjoint. The p values were corrected for multiple comparisons and considered significant for *p* < 0.05. Either the comparison intervals or the *p*-values are provided. Each timepoint for each ROI was compared to the other 3 timepoints. All error bars correspond to the standard error of the mean.

Full recovery was established when the two following conditions were fulfilled: (i) a significant difference was detected between the 12-week TX timepoint and either the 6-week or 12-week recovery timepoints and (ii) no significant difference was found between the pre-TX and the 6-week or 12-week recovery timepoints. Partial recovery was established when either or both of the following conditions were fulfilled: (i) a significantly low difference was detected between the 12-week TX timepoint and either the 6-week or 12-week recovery timepoint and (ii) no significant difference was found between the pre-TX and the 6-week or 12-week recovery timepoints; or when a significant difference was detected between both (i) the 12-week TX timepoint and the 6-week or 12-week recovery timepoints and (ii) between the pre-TX and the 6-week or 12-week recovery timepoints.

### Immunohistochemical assessment

Two mice were sacrificed (one at 12 weeks of TX and one at 12 weeks with no TX), and perfused intracardially with 4% paraformaldehyde in phosphate buffer saline. The extracted brains were then post-fixed overnight at 4°C in the same fixative, cryoprotected for several hours at 4°C in phosphate buffer saline containing 30% sucrose, and frozen in melting isopentane. The brains were then sliced in 20 μm thick sections with a Microm cryostat for myelin assessment with PLP/GFP (proteolipid protein/green fluorescent protein). Microscopic scans of whole sections (pixel size 0.25 μm^2^) were acquired with a Nanozoomer 2.0-RSslide scanner (Hamamatsu Photonics, Hamamatsu Japan). Co-localization of brain regions with MR images was performed using anatomical landmarks from similar slices.

## Results

### Signal ratios

Signal enhancement was clearly visible from the T_2_w images at 12 weeks of TX in rCC, CPu, EC, CP, cCC, TH, and S1 (Figure 1, middle column) and some recovery was also visible at 12 weeks with no TX (Figure 1, right column) compared to the pre-TX baseline signal (Figure 1, left column).

**Figure 2:**
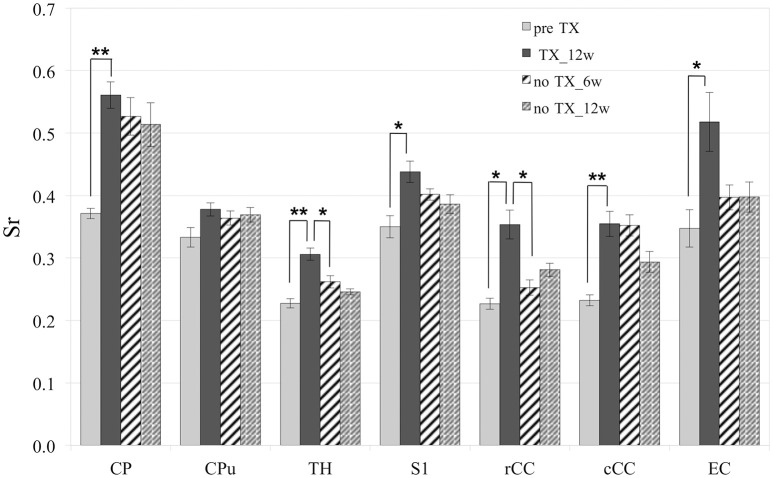
**Significant increase of Sr after 12 weeks (12 w) of TX in most structures**. Sr values came back to initial values after 6 weeks (6 w) or 12 weeks without TX in TH and rCC, partially in S1 and not in CP, Cpu, cCC, or EC. ^*^p < 0.05; ^**^p < 0.01.

Measurements of Sr after 12 weeks of TX showed significant enhancement in the superior CP (*p* < 0.006), TH (*p* < 0.005), S1 (interval [−15.6; −3.1]), rCC (*p* < 0.03), cCC (*p* < 0.004) and EC (*p* < 0.04) (Figure 2). After recovery (no TX) Sr fully came back to their initial values in TH (*p* < 0.005 at 12 weeks) and rCC (*p* < 0.03 at 6 weeks), only partially in S1 (interval [−0.6;13.8]; *p* < 0.2 at 12 weeks). No significant recovery of Sr was found in CP, CPu, cCC, or EC. However, no significant difference was found between pre-TX and no TX at 6 weeks in EC, which indicated partial recovery in this structure.

### T_2_ values

**Figure 3:**
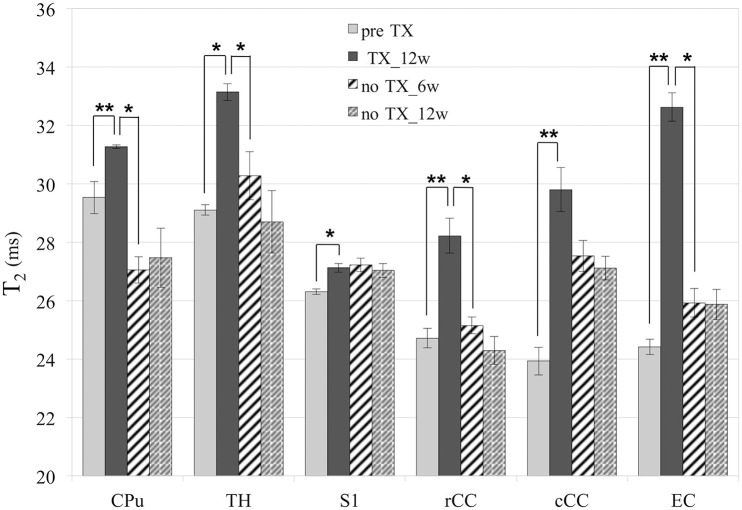
**T_2_ values**. Significant increase of T_2_ in all structures after 12 weeks (12w) of TX. Full or partial recovery after 6 weeks (6w) or 12 weeks without TX in all structures, except in S1. ^*^p < 0.05; ^**^p < 0.01.

T_2_ values (Figure 3) significantly increased in all GM and WM structures after 12 weeks of TX compared to pre-TX (CPu: *p* < 0.002; TH: *p* < 0.02; S1: *p* < 0.03; rCC: *p* < 0.01; cCC: *p* < 0.004; EC: *p* < 0.004). They then fully returned to initial values without TX in CPu (*p* < 0.04 at 12 weeks), in TH (*p* < 0.03 at 6 weeks), in rCC (*p* < 0.02 at 6 weeks) and in EC (*p* < 0.002 at 12 weeks). Only partial recovery was found in cCC as a significant difference was found both between the 12-week TX timepoint and the 12-week recovery timepoint, and between the 12-week TX timepoint and the pre-TX timepoint (*p* < 0.004). The T_2_ values measurements did not detect any recovery in S1 unlike Sr measurements.

### Diffusion

**Figure 4:**
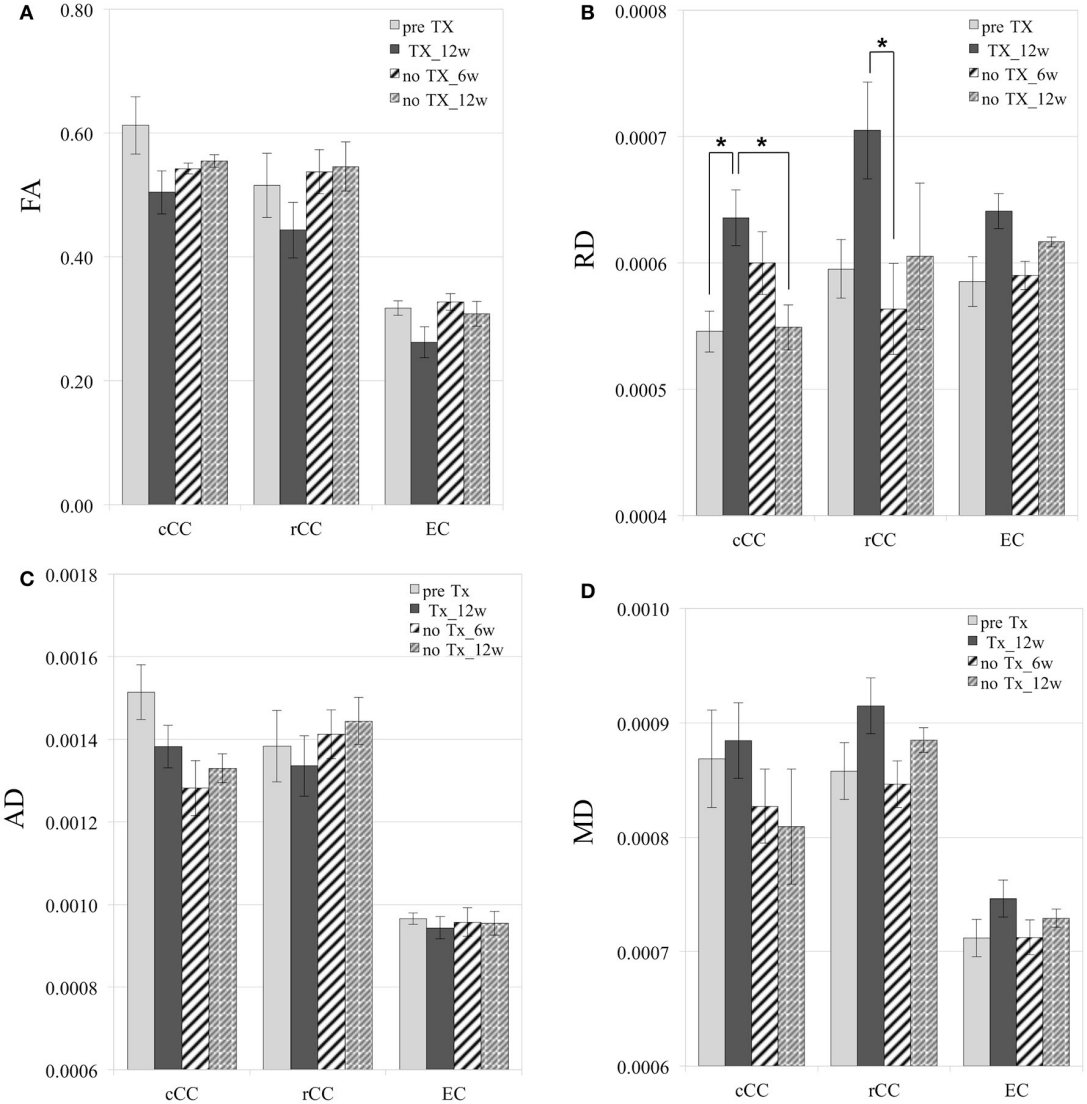
**Diffusion measurements**. Mild and reversible decrease of FA after 12 weeks (12 w) of TX **(A)**, although not significant. Reversible increase of RD in cCC, rCC, and EC **(B)**. No significant changes in AD **(C)**. Mild increase of MD at 12 weeks of TX and decrease at 6 weeks (6 w) without TX in rCC and EC **(D)**. RD, AD, and MD values are in mm^2^/s. ^*^p < 0.05.

After 12 weeks of TX, FA values decreased in all WM structures compared to preTX, and then increased back 6 weeks after recovery (Table 1). However, these changes were not significant due to large variability in the data (Figure 4A).

**Table 1:** **Diffusion metric values in white matter**.

	**FA**	**RD**	**AD**	**MD**
**PRE-TX**				
cCC	0.61 ± 0.07	0.00054 ± 0.00003	0.0015 ± 0.0001	0.00087 ± 0.00004
rCC	0.51 ± 0.08	0.00059 ± 0.00004	0.0014 ± 0.0001	0.00085 ± 0.00007
EC	0.31 ± 0.02	0.00058 ± 0.00003	0.0009 ± 0.00002	0.00071 ± 0.00003
**12-WEEK TX**				
cCC	0.50 ± 0.06	**0.00063** ± 0.00005^*^	0.0013 ± 0.0001	0.00087 ± 0.00006
rCC	0.44 ± 0.09	0.00070 ± 0.00009	0.0013 ± 0.0002	0.00088 ± 0.00005
EC	0.26 ± 0.04	0.00064 ± 0.00003	0.0009 ± 0.00006	0.00074 ± 0.00004
**6-WEEK NO TX**				
cCC	0.54 ± 0.05	0.00059 ± 0.00005	0.0012 ± 0.0001	0.00083 ± 0.00005
rCC	0.53 ± 0.08	**0.00056** ± **0.00008^#^**	0.0014 ± 0.0001	0.00082 ± 0.00006
EC	0.32 ± 0.03	0.00059 ± 0.00002	0.0009 ± 0.00007	0.00070 ± 0.00003
**12-WEEK NO TX**				
cCC	0.55 ± 0.02	**0.00055** ± **0.00005^#^**	0.0013 ± 0.00007	0.00081 ± 0.00002
rCC	0.54 ± 0.04	0.00060 ± 0.00011	0.0014 ± 0.00006	0.00083 ± 0.00003
EC	0.31 ± 0.04	0.00062 ± 0.000008	0.0009 ± 0.00003	0.00072 ± 0.000005

The values represent mean ± standard deviation;

* statistically different from pre-TX (p < 0.03);

# *statistically different from 12-week TX (p < 0.03). RD, AD, and MD values are in mm^2^/s. Significant values are in bold*.

RD values significantly increased 12 weeks after TX in cCC (*p* < 0.03), and non-significantly in rCC (*p* < 0.1) and in EC (*p* < 0.2) compared to preTX (Table 1). During the recovery phase, RD values fully returned to their initial values at 6 weeks in rCC (interval [1.1;13.8]), at 12 weeks in cCC (*p* < 0.03) and non-significantly in EC at 6 weeks (interval [−0.2;12.2]) (Table 1; Figure 4B). These RD results suggest clear demyelination during the TX phase in WM structures then remyelination during the recovery phase.

AD measurements showed no significant changes in rCC and EC (Figure 4C). In cCC, non-significant decrease of AD was measured at 12 weeks of TX, and at 6 weeks and 12 weeks without TX compared to before TX (Table 1), suggesting a progressive effect of the TX on AD values in cCC.

MD mildly increased in rCC and EC at 12 weeks of TX compared to before TX but not in cCC (Table 1, Figure 4D). After 6 weeks of recovery, MD values returned to initial values in rCC and in EC, however those results were not significant due to large variability in the data (Table 1).

In GM regions, no changes in diffusion metrics were detected.

### Immunohistochemical assessment

**Figure 5:**
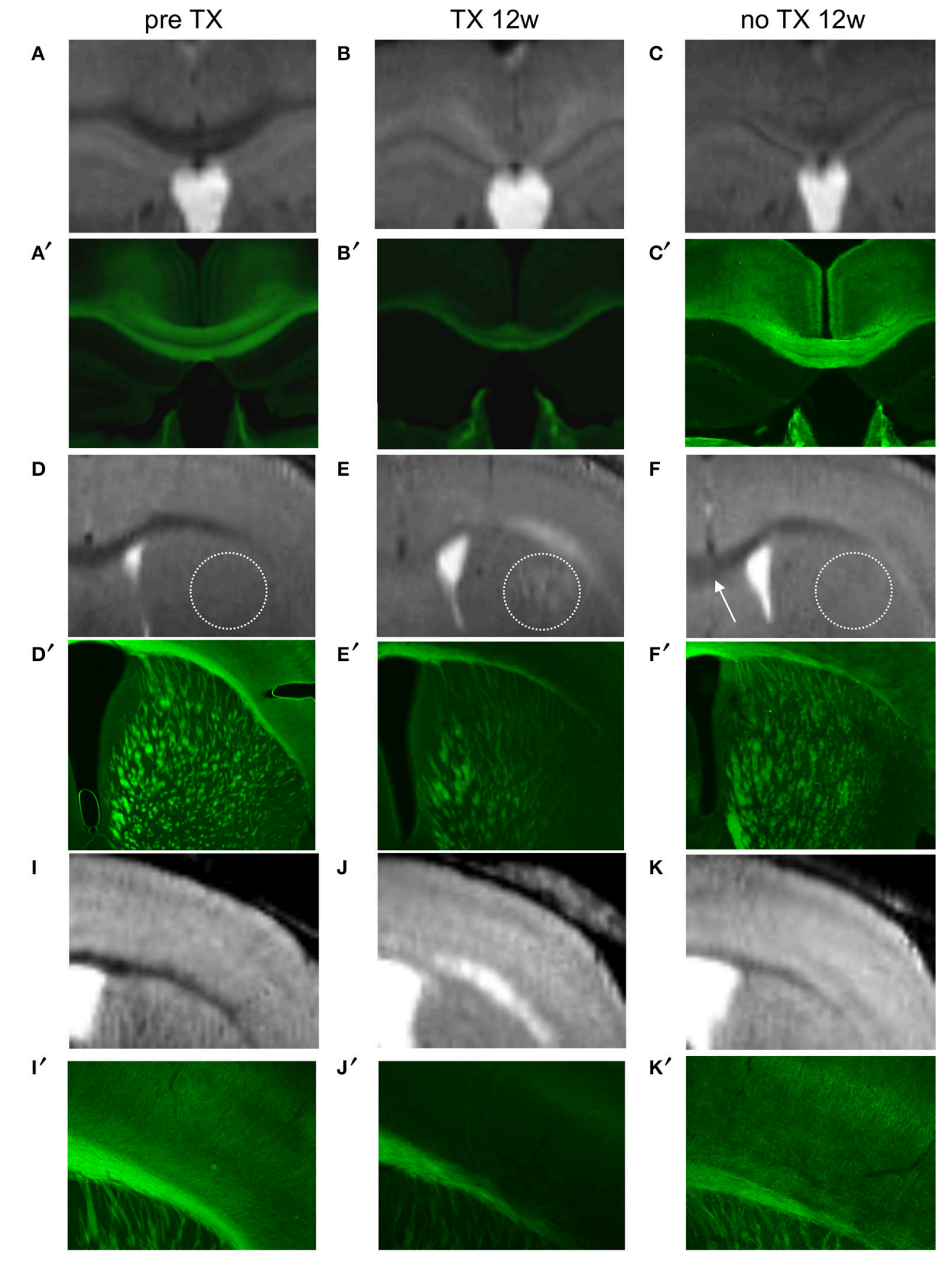
**Colocalization of demyelinated and remyelinated regions between MRI (A–K) and PLP/GFP immunohistochemical sections (A′–K′) before TX, 12 weeks (12w) after TX, and 12 weeks after TX removal**. The dotted circle represents CPu and the arrow points to rCC.

Immunohistochemical evaluation of coronal brain sections at 12 weeks of TX confirmed demyelination in CC, EC, CPu, TH, and in S1 cortex identified as decreased GFP fluorescence. After 12 weeks of recovery a clear but less bright fluorescence reappeared in the same regions reflecting myelin regeneration. However, at this stage we found heterogeneity in the fluorescence intensity, which was more extended and more intense in CC and EC and showed intermediate intensity in GM regions, especially in the cortex. ROIs analyzed on MRI were analyzed in parallel by immunohistochemistry (Figure 5), showing that MRI changes with time were associated with myelin regeneration in the same regions.

## Discussion

### Main results

We used the chronic CPZ demyelinating model and we showed that ultra-high-field MRI with a CryoProbe™ was sensitive for the detection and the quantification of demyelination and remyelination processes. Following the 12-week CPZ TX, T_2_w sequences, either by quantifying Sr or T_2_ values, could capture demyelination in WM areas such as in CC and in EC, but was also shown to be sensitive enough for measuring demyelination in the deep GM and in the cortex. Diffusion-weighted metrics were less sensitive to demyelination in the GM and could only quantify myelin loss and recovery (RD changes) in the main WM tracts (in accordance with already published data on the CPZ model treated for 6 weeks, Song et al., 2005; Zhang et al., 2012). When the neurotoxic agent was removed, a clear improvement of Sr and T_2_ value paralleling myelin regeneration on immunohistological samples was found both in the WM and in the deep GM, whereas in S1, only a mild trend to normalization of Sr was detected. In some regions, Sr and T_2_ values measurements showed varying levels of sensitivity in detecting demyelination (e.g., in CPu) or remyelination (e.g., in cCC or EC).

We also observed mild tremor and rigidity in mice under TX, which disappeared during recovery.

### Model considerations

In this chronic 12-week TX paradigm we showed that a significant level of myelin regeneration could indeed occur within the 12 weeks that follow the interruption of TX, contrary to the previous idea that longer CPZ exposure was associated with failing remyelination (Armstrong et al., 2006). However, under this condition the remyelination was overall suboptimal and delayed compared to the classical 6-week TX paradigm where a complete remyelination is expected to occur within 4–6 weeks. This confers a major interest for the paradigm we used here, as it reproduces more closely the *in vivo* condition observed in human diseases such as MS, where remyelination is quite slow and incomplete. This system could provide an opportunity to better investigate candidate promyelinating drugs in this experimental model not only for their potential to accelerate the process of remyelination, but also for their ability to enhance the amount of myelin regeneration in unfavorable conditions.

Our results also indicate that the sensitivity of ultra-high-field MRI could be of interest to investigate myelin dynamics in GM regions, as a significant recovery was observed in deep GM regions together with a trend toward normalization of Sr in S1. Demyelinating lesions in the MS cortex were identified as strongly associated with physical and cognitive disability in MS (Calabrese et al., 2009), suggesting that their potential to remyelinate could be a crucial prognosis factor. On the other hand, pathological investigations have well attested that even in late stages of the disease, these lesions may retain a significant ability to spontaneously regenerate myelin (Chang et al., 2012). Therefore, the identification of putative promyelinating therapies positively acting on cortical remyelination has recently emerged as one of the key objective to reach in the regenerative approach for MS. The imaging methodology we used in the CPZ model may enable selection of candidate drugs depending on their impact on cortical repair. The smaller amount of myelin contained in the cortex compared to WM drastically decreases the sensitivity of the T_2_w sequence to myelin content changes, explaining why the recovery did not reach statistical significance using T_2_ measurements in this limited sample of animals. In addition, the *ad libitum* intake of CPZ could account for the large variability between mice, although the animals' weights were fairly similar. Further studies should include larger samples (however 5–8 mice were also used in most other similar studies, Song et al., 2005; Zhang et al., 2012) and may allow the detection of significant changes, especially when cortical remyelination is pharmacologically enhanced.

### Imaging considerations

The use of both a very high magnetic field (11.7T) and a Cryoprobe in this study enabled very high resolution with high SNR in the T_2_w sequence with a voxel volume of 0.8 × 10^−3^ mm^3^ acquired in only 17 min, compared to voxel volumes used in similar studies with lower fields and conventional probes: from 1.6 × 10^−3^ mm^3^ at 2.35T (scan time not provided) (Merkler et al., 2005), to 3 × 10^−3^ mm^3^ in 20 min at 9.4T (Zhang et al., 2012), and to 7 × 10^−3^ mm^3^ in 10 min (Thiessen et al., 2013) and 10 × 10^−3^ mm^3^ (scan time not provided) (Chandran et al., 2012) at 7T. Our voxel volume was 2 to more than 10 times smaller than in other studies with comparable scan times, which conferred to our T_2_w protocol an increased sensitivity to detect signal changes in numerous WM and GM brain regions.

Although a disadvantage of T_2_w sequences could have been their non-specificity to a particular type of brain lesion, these sequences are highly sensitive, especially at very high resolutions (60 μm). In the model used, the requirement for sensitivity primed over specificity as the underlying pathology at the time that it was investigated and mainly consisted of myelin dynamic changes, with no edema or blood-brain barrier leakage, and only a minor inflammatory component as the microglial reaction predominates between the 3rd and the 5th week of TX and subsequently decreases in this model (Skripuletz et al., 2011).

Other sequences such as diffusion or magnetization transfer imaging were described as more specific for myelin compared to T_2_w sequences, however they suffer from lower spatial resolution, less reproducibility and worse SNR. While these sequences are of great interest for the quantitative investigation of the myelin compartment, they may be less sensitive to mild or moderate myelin dynamic changes, as attested by the negative results described in this study for diffusion parameters in the GM. Along the same line of results, magnetization transfer imaging showed some changes following the CPZ TX in the deep GM, but failed to detect any change in the cortex (Fjær et al., 2013).

### Methodological considerations

Although T_2_ values are known to shorten with aging in normal mouse brains from about −0.05 to −0.1 ms/100 days as measured by Falangola et al. at 7T (Falangola et al., 2007), no significant changes in C57Bl6 WT mice were expected throughout our study as shown by Sun et al. MRM 2006 who also used 8-week old C57Bl6 mice for the first timepoint with a similar experimental protocol (longitudinal follow-up from 12-week of TX to 12-week of recovery, 4.7T). We calculated the estimated mean T_2_ values from those rates of changes for the control group over all timepoints. We found that the maximum percent change in T_2_ was −0.66% by the last timepoint (168 days later). From our data, we were able to detect significant T_2_ changes in the order of 3.2–30% between timepoints, which was 5–50 times larger than the estimated T_2_ changes of the control group over time. We can therefore consider that the significance of our T_2_ results would have remained unchanged even with an additional age-matched control group at each timepoint. Interestingly, a recent study (Jelescu et al., 2016) showed T_2_ reductions in the order of 8% over an 18-week period in the CC of similar control mice. These results suggest that T_2_ shortens more in regions highly dense in myelinated WM than regions poorly dense in WM as shown in Falangola's study.

Our analysis method was based on manually drawn ROIs and did not allow for systematic evaluation of the entire brain, which explains why the cerebellum and the cortex were not thoroughly examined so that more lesions could potentially be detected (e.g., in the cerebellar WM). However, automatic or semi-automatic measurement methods based on normalized ROIs on a template have limitations for very small regions such as CC or EC: errors in measurements can arise from misaligned ROIs and subsequent manual adjustments are necessary and time-consuming.

### Perspectives

Overall this work emphasizes that ultra-high field MRI is sensitive enough to quantify dynamic demyelination and remyelination in the chronic CPZ model both in the WM and in the GM. This opens the perspective to investigate promyelinating compounds *in vivo* for their ability to promote repair in both regions, a step that will be crucial for the selection of molecules that should be further developed in early clinical trials in MS.

## Author contributions

All authors substantially contributed to the study. AP performed the data acquisition and analysis and wrote the manuscript, MA performed the immunohistochemistry and wrote the manuscript, BS designed the study and wrote the manuscript.

### Conflict of interest statement

The authors declare that the research was conducted in the absence of any commercial or financial relationships that could be construed as a potential conflict of interest. The reviewer FG and handling Editor declared their shared affiliation, and the handling Editor states that the process nevertheless met the standards of a fair and objective review.

## Abbreviations

CPZ: cuprizone
TX: treatment
WM: white matter
GM: gray matter
CC: corpus callosum
cCC: caudal corpus callosum
rCC: rostral corpus callosum
CP: cerebellar peduncle
EC: external capsule
CPu: caudate putamen
TH: thalamus
Sr: signal ratio.
